# Analysis of selected diploid banana genotypes for resistance to weevil damage and pollen quantity as key elements of banana breeding

**DOI:** 10.3389/fpls.2025.1620276

**Published:** 2025-09-25

**Authors:** Juliet Kemigisa, Ivan Kabiita Arinaitwe, Jerome Kubiriba, Arthur K Tugume, Robooni Tumuhimbise

**Affiliations:** ^1^ National Banana Research Programme, National Agricultural Research Laboratories, Kampala, Uganda; ^2^ Department of Plant Sciences, Microbiology and Biotechnology, College of Natural Sciences, Makerere University, Kampala, Uganda

**Keywords:** diploid bananas, pollen quantity, banana weevils, resistance, breeding

## Abstract

Banana weevils (*Cosmopolites sordidus*) cause significant reductions in banana productivity in Uganda. Their distribution extends countrywide, with higher concentrations in the central region due to favorable environmental conditions. Integrated weevil management practices incorporate resistance into susceptible genotypes through breeding, which utilizes pollen from resistant diploid bananas. Field and pot screening experiments were conducted in central Uganda (Kawanda) to assess the response of nine outsourced diploid banana genotypes from the International Musa Transit Centre (ITC) to weevil damage. Pollen quantity of the bananas was also evaluated. The percentage of weevil damage on the peripheral and cross sections of the corms was recorded. Pollen quantity was scored on a scale of 0 to 4, with 0 representing no pollen and 4 the highest pollen production. Results showed that the genotypes Saing hil, Pisang gigi buaya, Pisang rotan, Pisang tunjuk, Morong princessa, Morong datu, and Gabah gabah were resistant to weevil damage compared to the susceptible genotypes Nakitembe and Kibuzi (EAHB). Saing hil and SH-3142 exhibited higher pollen quantities of 3.4 and 3.0, respectively, which were closest to the value of 4 observed in ‘Calcutta 4’, the most male-fertile wild diploid. Saing hil combined high resistance to weevil damage with high pollen quantity and is therefore recommended for use in conventional banana breeding.

## 1 Introduction

Bananas (*Musa* spp.) are an important staple and livelihood crop for over 70 million people in Africa (Voora et al., 2020). They are processed into products such as canned banana slices, crisps, jam and jelly, medicine, banana flour, and powder to serve different consumption purposes (Abiodun-Solanke and Falade, 2011). In East Africa, traditional triploid clones of the East African Highland bananas (EAHBs) dominate other genotypes (Marimo et al., 2019). In Uganda, most households depend on EAHBs and dessert bananas for food security, household income, and cultural values (Williamson, 2021; Lee, 2023). Despite the growing demand for food in Uganda, banana production has declined from 10 metric tonnes/ha/yr in 2000 to 4 metric tonnes/ha/ya (FAO, 2021). This decline is attributed to both abiotic and biotic stresses, which include depleting soil nutrients, primitive agronomic practices, parasitic nematodes, banana weevils, and diseases such as black Sigatoka, fusarium wilt, and banana xanthomonas wilt (Nyombi, 2013; Bwogi et al., 2014).

The banana weevil (*Cosmopolites sordidus* Germar) causes estimated yield losses of 40%–60% and, if uncontrolled, reduces plantation lifespan in severe cases (Gold et al., 2004; Nyombi, 2010; Oliveira et al., 2017). Weevil damage occurs on the corm through larval tunneling, which creates entry passages for parasitic fungi, interferes with root system development, reduces yields, and increases mat disappearance (Kiggundu et al., 2003b; Gold et al., 2006; Arinaitwe et al., 2014). Current management interventions rely on cultural practices such as the use of clean planting materials, systematic trapping and killing of adult weevils, and field hygiene (Erima et al., 2016). Chemical control using synthetic insecticides and pheromones is a common practice among large-scale banana producers in Africa (Gold et al., 2001). In some countries, myrmicine ants (*Tetramorium guinense*, *Pheidole megacephala*) are deployed to feed on weevil larvae (Gold and Messiaen, 2000). Other biological measures employ entomopathogenic fungi such as *Beauveria bassiana* and *Metarhizium anisopliae* (Tinzaara et al., 2015), which break down the weevil exoskeleton and cause death. However, these interventions have not been sustainable, as they increase production costs for small-scale farmers, and weevils eventually re-infest plantations (Omukoko et al., 2014).

The most promising and reliable method of managing banana weevils is the deployment of resistant banana varieties (Kiggundu et al., 2003; Arinaitwe et al., 2014; Twesigye et al., 2018). To address declining banana production in Uganda, the National Banana Research Program of the National Agricultural Research Organization (NARO) is continuously developing and disseminating high-yielding and resistant banana hybrids to supplement the highly susceptible EAHBs (Nowakunda et al., 2015; Tumuhimbise et al., 2018). Over the years, eight banana hybrids tolerant to weevils have been developed and promoted among farming communities for adoption in banana-based farming systems (Nowakunda et al., 2015; Tumuhimbise et al., 2018). Consequently, banana varieties, namely, KABANA 6H, KABANA 7H, NAROBan1, NAROBan2, NAROBan3, NAROBan4, NAROBan5, and NAROBan6, were conventionally bred, released, and added to the national cultivar list in Uganda.

The main source of resistance to banana pests and diseases is harnessed from wild banana relatives, diploids, and improved diploids generated from intentional crosses (Jain and Priyadarshan, 2009). These are crossed with susceptible banana triploids to introgress traits of interest and develop preferred banana hybrids. However, diploid bananas are not indigenous to Uganda and are mostly sourced from centers of origin or the International Musa Germplasm Transit Centre (ITC). The Uganda national banana breeding program has sourced diploids from ITC to integrate into breeding schemes and to increase the genetic base of previously acquired diploids. However, the breeding value of these sourced diploids in terms of resistance to weevils, agronomic performance, and pollen availability remained unclear. Therefore, this study aimed to screen the outsourced banana diploids to determine their resistance to banana weevil, their agronomic performance, and their pollen quantity as a useful resource in conventional breeding.

## 2 Materials and methods

### 2.1 Site description

Field and screen house experiments were carried out at the National Agricultural Research Laboratories (NARL) research station, Kawanda, located in Wakiso District at an elevation of 1,195 m above sea level (0°25’N, 32°32’E). Average daily temperatures are 15 °C (minimum) and 29 °C (maximum), with a mean relative humidity of 76%. Kawanda receives a mean annual rainfall of approximately 1,189 mm in a bimodal distribution between March–June and September–December.

### 2.2 Experimental materials

Thirteen banana genotypes, which included nine banana diploids sourced from the International Musa Germplasm Transit Centre (ITC) as *in vitro* proliferating tissues and four reference (check) genotypes obtained from the NARL plantations in Kawanda, were used in this study. The diploids sourced from ITC belong to the ‘Pisang Jari Buaya’ family, and their selection was based on reported resistance to nematodes (Wehunt and Hutchison, 1978). These diploids are cultivated, except for SH-3142, which is used for breeding purposes only. The four reference genotypes included Calcutta 4 and Kayinja as resistant controls to weevils, and Kibuzi and Nakitembe (EAHB clones) as susceptible controls (Shukla, 2010) (**Table 1**). Tissue culture plantlets (Al-Amin et al., 2009; Van, 2009) of the banana genotypes were assessed both in the field and in the screen house for weevil damage. The screen house experiments were carried out between October 2017 and April 2018, and the field experiments between August 2016 and March 2019.

**Table 1:** Characteristics of banana genotypes used in the study.

S/N	Genotype	Ploidy	Response to *Radopholus similis*	Response to weevils	Reference
1	Morong Datu	AA	Partially resistant	Not known	Wehunt and Hutchison, 1978
2	Pisang Gigi Buaya	AA	Resistant	Not known	Wehunt and Hutchison, 1978
3	Pisang Tunjuk	AA	Resistant	Not known	Wehunt and Hutchison, 1978
4	SH-3142	AA	Resistant	Not known	Wehunt and Hutchison, 1978
5	Pisang Rotan	AA	Partially resistant	Not known	Wehunt and Hutchison, 1978
6	Huwundu vita	AA	Resistant	Not known	Wehunt and Hutchison, 1978
7	Gabah Gabah	AA	Resistant	Not known	Wehunt and Hutchison, 1978
8	Saing- Hil	AA	Resistant	Not known	Wehunt and Hutchison, 1978
9	Morong Princessa	AA	Resistant	Not known	Wehunt and Hutchison, 1978
10	Calcutta 4	AA	Resistant	Resistant check	Sadik et al., 2010
11	Kayinja	ABB	Resistant	Resistant	Kiggundu et al., 2006
12	Kibuzi (EAHB)	AAA	Susceptible	Susceptible check	Shukla, 2010
13	Nakitembe (EAHB)	AAA	Susceptible	Susceptible check	Shukla, 2010

### 2.3 Trappings, rearing, and sexing weevils

The weevils used in the experiment were trapped in old banana plantations at Kawanda using the method described by Juliana et al. (2017). They were reared in plastic buckets and fed with detached corms of Kibuzi (EAHB) for 1 week. Visible marks on the lower abdominal segment (rostrum) of the weevils were used to determine sex, as described by Longoria (1968).

### 2.4 Experimental design

#### 2.4.1 Screen house experiment

**Figure 1:**
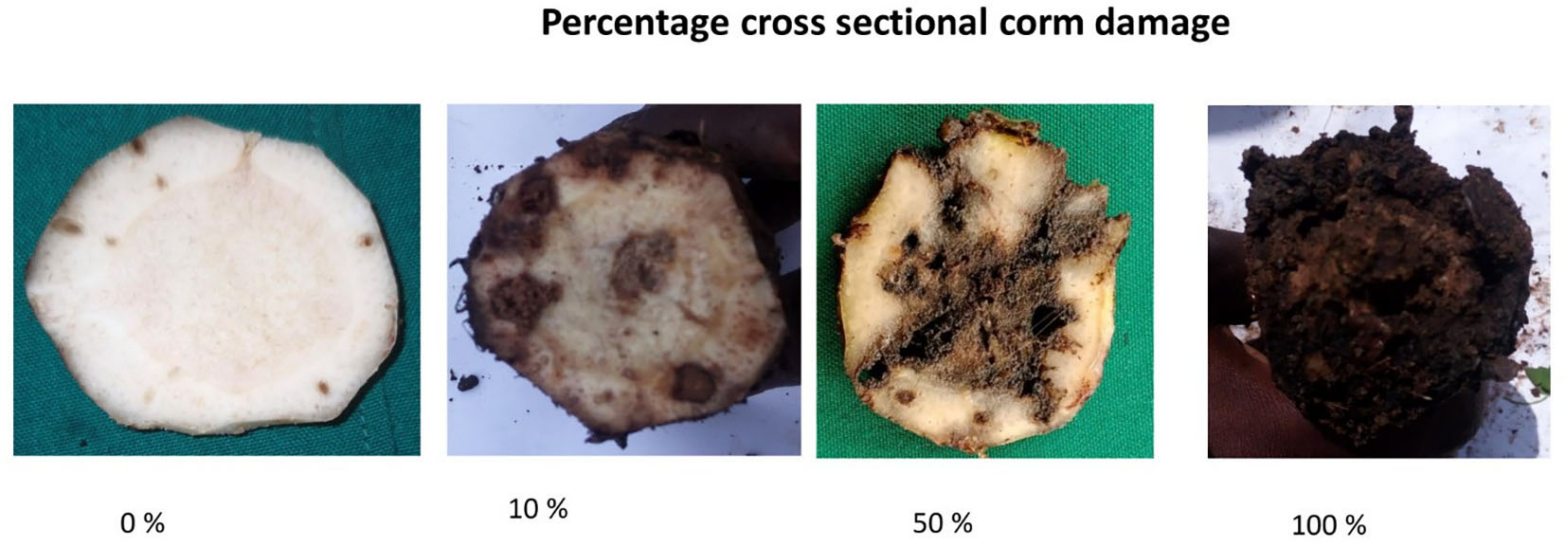
Different cross sectional corm damage caused by banana weevils after 60 days of infestation.


The pot experiment was conducted using the method described by Sadik et al. (2010) with modifications, including the use of 10 L buckets and reducing the number of weevils to six. Ten genotypes (**Table 1**), including nine diploids and one East African Highland Banana (EAHB), were hardened in 10 L plastic buckets filled with sterilized loam soil, sand, and decomposed farmyard manure at a ratio of 3:1:1 for 5 months. The experiment was set up in a randomized complete block design with five replicates. Each replicate contained at least three clones of the same genotype randomly placed within the block. After 5 months of hardening and attaining the desired corm size, the plants were infested with six sexed adult weevils (three females and three males) placed at the base of each plant. To prevent escape, the weevils were restrained by sealing the pots with weevil-proof nylon nets. Sixty days after infestation, the experiment was terminated by uprooting the plants from the pots. Corm damage assessment was carried out by visually observing the galleries created by the larvae. Damage was assessed according to Gold et al. (2005) at three points: (i) on the periphery of the peeled corm, (ii) on the cross section cut at 3 cm, and (iii) on the cross section cut just below the collar region (6 cm). Peripheral damage was estimated as the percentage surface damage after peeling off roots and removing soil. Transverse cross sections at the collar region and 3 cm below revealed the extent of weevil galleries. Percentage damage of the corm circumference was estimated for both the inner and outer cortex at each level (**Figure 1**). The overall effect of weevils on the corm was obtained by computing the average damage from the peripheral and cross-sectional assessments. Numbers of adult weevils and larvae recovered from pots were also recorded.

#### 2.4.2 Field experiment

Two-month-old hardened tissue culture plantlets of 12 genotypes were planted in holes measuring 45cm x 45cm in diameter and depth. Each hole was filled with 5 kg of decomposed farmyard manure mixed with topsoil before planting at the recommended spacing of 3m x 3m = 9 m² per plant). The experimental field included five replicates, each with at least four clones per genotype, for a total of 240 banana mats. The design used was a randomized complete block design (RCBD). Border rows were planted with Yangambi KM 5, a weevil-resistant variety. Field management included periodic hand weeding, de-suckering, maintaining plant density at three plants per mat, and de-leafing (removal of old and dry leaves).

##### 2.4.2.1 Weevil infestation in the field experiment

The experiment was planted in a weevil hot spot area to allow continuous exposure of the genotypes to weevils throughout their growth cycle. At nine months after planting, weevil density in the experiment was increased by manual infestation with 10 adult weevils (five males and five females) released at the base of each banana mat. Infestation was carried out between 5:00 and 6:00 p.m. to avoid loss through desiccation in the afternoon sun. This approach was intended to distribute banana weevils evenly across the plantation and supplement natural infestation.

### 2.5 Data collection

#### 2.5.1 Agronomic and yield data

Agronomic data on plant performance indicators—including plant height (PH), plant girth (PG) at 1 m from the ground, number of functional leaves (NFL), and height of the tallest sucker (HTS)—were recorded at flowering for two crop cycles (Gaidashova et al., 2010; Melo and Coelho, 2016). Plant height was measured using a calibrated wooden stick (precision ±1 cm) from soil level up to the intersection of the petioles of the two youngest leaves. Plant girth was measured using a tailor’s measuring tape (precision ±0.5 cm). Functional leaves were defined as those with ≥50% green surface area. Plant stature was calculated as the ratio of plant height to plant girth. The maturity period was measured as the time from flowering to harvest. A banana bunch with a ripening finger was considered mature and ready for harvest. Yield performance indicators recorded at harvest included bunch weight (kg), measured using a hanging balance (precision ±0.5 kg), number of clusters per bunch, number of fingers per bunch, finger circumference (cm), and finger length (Gaidashova et al., 2010; Kamira et al., 2016; Melo and Coelho, 2016). Fingers were sampled from the second cluster from the bottom and from the topmost cluster of the bunch. Finger length and circumference were measured using a tailor’s measuring tape (precision ± 0.5 cm).

#### 2.5.2 Pollen quantity

Pollen was quantified for the first cycle only. After the first cycle, the male buds where pollen was to be collected were removed prematurely as a phytosanitary control measure for banana bacterial wilt, which had become rampant in the experimental trial. Eight to ten anthers were cut from the relatively newly opened bracts (banana floral structures) of each genotype and rated for pollen quantity on a scale of 0 to 4 (0 = no pollen, 1 = very little pollen, 2 = little pollen, 3 = moderate pollen, 4 = abundant pollen like Calcutta 4). Anthers were brushed on the back of the hand, and trained personnel subjectively rated the amount of pollen that dropped out according to the scale.

#### 2.5.3 Weevil damage

Weevil damage was assessed on uprooted corms of harvested and toppled flowered bananas within 10 days after harvest or toppling, respectively. Percentage weevil damage was scored on the periphery and on cross sections at the collar region and 5 cm below the collar region (Gold et al., 2005). Peripheral damage was estimated after paring the outer roots and soil to reveal the weevil galleries created by the larvae. Peripheral damage was expressed as the percentage corm surface damage. Cross-section damage was assessed on two regions of the corm that were cut transversely to reveal weevil galleries. Percentage cross-section damage was estimated by first dividing the corm circumference into four equal quarters (25%). These were further divided into halves or quarters to give the true representation of the damage in that section. Percentage cross-section corm damage was the sum of the damage in the four quarters. The first cross section was made at the collar region, and the percentage weevil galleries of the corm circumference were estimated for the inner and outer cortex. Cross section two was made 5 cm below the collar region, and the percentage weevil damage of the transverse corm circumference was recorded for both the inner and outer cortex. The total cross-section weevil damage (TXD) was the sum of the inner and outer cortex (**Figure 1**). The total weevil damage (TD) on the corm was computed from **Equation 1** below. Weevil damage assessment was done on plants in the first and second cycles. Other major weevil effects, such as mat disappearance and snapping before flowering or harvest, were also monitored.

(1) $$TD=\frac{PD+TXD}{2}$$

where TD is total damage, PD is peripheral damage, and TXD is total cross section damage.

### 2.6 Data analysis

Statistical analysis was done using IBM SPSS (Williamson, 2021) statistics version 20. Genotype means were compared using one-way analysis of variance (ANOVA). Means were separated using Games–Howell at P<0.05. Correlations between weevil damage in the pot experiment were measured using Spearman’s correlation coefficient. Pearson’s correlation coefficient was computed to assess the relationship between weevil damage in the screen house and field experiments for the genotypes evaluated in both.

## 3 Results

### 3.1 Variability of the genotypes for weevil damage parameters

All genotypes expressed highly significant differences (P<0.001) for weevil damage at the periphery, cross section, overall total damage, and larvae recovered in pots, whereas adult weevils were not significant (P<0.14). Mean overall weevil damage (total damage) ranged from 12.7% for Calcutta 4 (resistant check) to 81.8% for Nakitembe (EAHB, susceptible check) in the pot experiment (**Table 2**). The EAHBs showed the highest weevil damage at both the periphery and cross section, indicating that they were most palatable for weevils. Generally, the periphery of the corm was more damaged than the cross section in both pot and field experiments, indicating that damage progressed from the outer cortex after eggs hatched into the inner cortex. Mean overall weevil damage was significantly lower for all diploids than for the susceptible EAHB control, and comparable to Calcutta 4. This classified all diploids as resistant and EAHB as susceptible.

**Table 2:** Weevil damage indicators of genotypes evaluated under screen house conditions.

Genotype	Number of larvae ± se	Number of weevils ± se	Peripheral damage (%) ± se	Cross section damage (%) ± se	Total damage (%) ± se
Morongo Datu	0.9 ± 0.2ab	0.4 ± 0.3	33.7 ± 8.7a	35.8 ± 7.6a	35.3 ± 7.8a
Pisang Gigi Buaya	1.3 ± 0.3ab	0.0 ± 0.0	40.4 ± 8.8a	38.0 ± 6.1a	38.5 ± 6.5a
Pisang Tunjuk	0.7 ± 0.3ab	0.0 ± 0.0	16.7 ± 6.9a	12.8 ± 4.2a	13.5 ± 4.6a
SH-3142	0.9 ± 0.5ab	0.2 ± 0.1	59.4 ± 9.2b	50.8 ± 10.1ab	52.5 ± 9.8ab
Pisang Rotan	0.6 ± 0.4ab	0.0 ± 0.0	38.4 ± 13.4ab	31.4 ± 14.5ab	32.8 ± 14.7ab
Huwundu Vita	1.5 ± 0.3b	0.7 ± 0.3	43.3 ± 9.8a	39.8 ± 10.1a	40.5 ± 10.0a
Gabah Gabah	1.0 ± 0.3ab	0.2 ± 0.1	51.4 ± 10.0ab	44.3 ± 9.8ab	45.8 ± 10.0ab
Saing Hil	0.9 ± 0.3ab	0.3 ± 0.1	45.3 ± 6.9a	22.8 ± 5.2a	27.3 ± 5.2a
Calcutta 4	0.3 ± 0.1a	0.1 ± 0.1	15.0 ± 8.3a	12.1 ± 7.4a	12.7 ± 7.8a
Nakitembe-EAHB	2.6 ± 0.8ab	0.1 ± 0.1	83.8 ± 4.2b	81.3 ± 5.6b	81.8 ± 5.1b
Mean	1.03 ± 0.1	0.2 ± 0.1	41.5 ± 3.1	35.5 ± 2.9	36.7 ± 2.9
F(9,127)	2.962	1.544	4.706	5.493	5.395

Data represent means from the assessed genotypes with their standard error. Means with the same letters within column are not significantly different (P<0.05). se, standard error of the mean, EAHB-East African High land banana.

Mean overall weevil damage for SH-3142, Gabah gabah, and Pisang rotan was not significantly different from Calcutta 4 and Nakitembe in the pot experiment. A clear distinction of genotypes’ weevil damage was expressed in the field experiment, where SH-3142 was more damaged and distinguishable from both Calcutta 4 and the EAHBs. Genotype SH-3142 was therefore classified as tolerant. The susceptible EAHB recorded the highest number of larvae (2.6), followed by Huwundu vita (1.5). The same genotypes had 0.1 and 0.7 adult weevils, respectively, representing the lowest and highest counts. The number of adult weevils in all genotypes was less than six, the initially infested number, indicating weevil mortality after infestation and oviposition. Overall, weevil damage in the pot experiment ranged from 12.7% to 81.8%, whereas damage ranged from 0.2% to 17.4% in the field experiment. Higher damage in pots was attributed to smaller corm size and confined weevils, unlike the field where damage was estimated on larger corms. Under field conditions, weevils may exhibit antixenosis.

Weevil damage from the field screening experiment is presented in **Table 3**. Genotypes Pisang gigi buaya, Pisang rotan, Pisang tunjuk, Morong princessa, Morong datu, Huwundu vita, Saing hil, and Gabah gabah exhibited high levels of resistance to weevils similar to Calcutta 4, with damage <2. The overall, cross-sectional, and peripheral weevil damage of these diploids was significantly different from the susceptible reference genotype (EAHB) in the field experiment, whereas there was no significant difference from Calcutta 4 and Kayinja (resistant) (**Table 3**). The mean peripheral corm damage of most genotypes was greater than cross-sectional damage in both pot and field experiments. These results confirmed the susceptibility of EAHB genotypes and the high resistance of all diploids evaluated.

**Table 3:** Percentage weevil damage on different genotypes evaluated under field conditions.

Genotype	Peripheral damage ± se	Cross-sectional damage ± se	Total damage ± se
Morongo datu	5.9 ± 2.2 a	0.7 ± 0.4 a	1.8 ± 0.7 ab
Pisang gigi buaya	0.6 ± 0.3 a	0.3 ± 0.2 a	0.4 ± 0.2 ab
Pisang tunjuk	1.1 ± 0.3 a	0.3 ± 0.1 a	0.4 ± 0.1 ab
SH-3142	4.7 ± 1.2 a	1.5 ± 0.5 a	2.2 ± 0.5 b
Pisang rotan	0.5 ± 0.3 a	0.3 ± 0.2 a	0.3 ± 0.2 a
Huwundu vita	2.5 ± 0.8 a	0.7 ± 0.3 a	1.1 ± 0.4 ab
Gabah gabah	2.0 ± 0.5 a	1.1 ± 0.4 a	1.3 ± 0.4 ab
Saing hil	1.0 ± 0.3 a	0.2 ± 0.1 a	0.3 ± 0.1 ab
Morongo princessa	1.3 ± 0.5 a	0.3 ± 0.1 a	0.5 ± 0.2 ab
Calcutta 4	0.7 ± 0.3 a	0.1 ± 0.0 a	0.2 ± 0.1 a
Kayinja	2.0 ± 0.5 a	0.5 ± 0.2 a	0.9 ± 0.2 ab
EAHB	31.9 ± 3.8 b	13.8 ± 2.4 b	17.4 ± 2.5 c
Mean	4.23 ± 0.6	1.6 ± 0.3	2.13 ± 0.3
F(11,321)	50.322	27.982	40.677

Data represent means from the assessed genotypes with their standard error. Means with different letters within a column are significantly different at (P<0.001), se, Standard Error of the mean; EAHB, East African Highland banana.

### 3.2 Spearman correlations for the weevil damage traits in the screen house experiment

The number of weevil larvae recovered in pots was positively and significantly correlated with all traits evaluated, except for the number of adult weevils recovered in pots, which was non-significant (**Table 4**). The highest correlation with larvae was recorded for cross-sectional and overall corm damage (r = 0.46), followed by peripheral damage (r = 0.41). The lowest correlation was recorded for adult weevils recovered in pots (r = 0.12).

**Table 4:** Correlations for the weevil damage traits obtained from banana genotypes evaluated in the pot experiment.

Traits	No. of larvae	No. of weevils	Peripheral damage	Cross section damage	Total damage
No. of larvae	1.00				
No. of weevils	0.12				
Peripheral damage	0.41***	0.27**			
Cross section damage	0.46***	0.30**	0.90***		
Total damage	0.46***	0.30***	0.94***	1.00***	1.00

**P<0.01, ***P<0.001.

Total corm damage was positively and significantly correlated (P < 0.001) with all traits evaluated. The highest correlation with total corm damage was recorded for cross-sectional damage (r = 1.0), and the lowest for adult weevils recovered in pots (r = 0.3).

### 3.3 Pearson correlation analysis for weevil damage in the screen house and field experiments

Correlation analysis of the weevil damage traits in the screen house and field experiments is presented in **Table 5**. Generally, most of the traits evaluated in both experiments were strongly and positively correlated. Cross-sectional damage in the field experiment was highly and significantly correlated with cross-sectional damage (r = 0.82) and total damage (r = 0.81) in the screen house experiment. Similarly, total damage in the field experiment was highly and significantly correlated with cross-sectional damage (r = 0.74) and total damage (r = 0.72) in the screen house experiment.

**Table 5:** Pearson correlation matrix of the results in the pot and field experiments.

Variables	Peripheral damage (pot)	Cross section damage (pot)	Total damage (pot)
Peripheral damage (Field)	0.30	0.57	0.55
Cross section damage (Field)	0.59	0.82**	0.81**
Total damage (Field)	0.49	0.74*	0.72*

*P <0.05, **P< 0.01.

By contrast, peripheral damage in both experiments recorded low and non-significant correlations with other weevil damage traits.

### 3.4 Performance of the genotypes for growth parameters at flowering and harvest

There were significant variations (P < 0.001) for all agronomic traits evaluated at flowering and harvest within diploids (AA) and triploids (ABB and AAA genomes). Averaged across banana growth cycles, diploid Saing hil recorded the largest plant stature (0.19), followed by Calcutta 4 and Kayinja (0.18), whereas Pisang rotan recorded the lowest (0.13) (**Table 6**). Saing hil had the shortest period between flowering and harvest (110 days), nearly two months earlier than Calcutta 4. The EAHB reference genotype Kibuzi was the first to reach harvest (102 days), followed by Saing hil (110 days), whereas Calcutta 4 was the most delayed (168 days). Genotype Kayinja (ABB) had the highest number of functional leaves at flowering (13.6), followed by Saing hil and Morong princessa (10.1), while Kibuzi had the lowest (7.2). The mean number of functional leaves for Calcutta 4 was significantly different from most genotypes, except Pisang rotan, SH-3142, and Kibuzi.

**Table 6:** Agronomic performance of different genotypes at flowering.

Genotype	Plant stature ± se	Number of functional leaves ± se	Height of the tallest sucker ± se	Days between flowering and maturity ± se
Morong datu	0.16 ± 0.00 b	7.6 ± 0.3 ab	199.3 ± 36.9 ab	122.0 ± 2.3 b
Pisang gigi buaya	0.14 ± 0.00 ab	8.8 ± 0.2 b	174.9 ± 17.6 a	134.5 ± 2.7 b
Pisang tunjuk	0.14 ± 0.01 ab	9.0 ± 0.2 b	189.2 ± 12.2 a	129.7 ± 2.7 bc
SH-3142	0.14 ± 0.00 ab	7.5 ± 0.3 a	199.4 ± 13.0 ab	141.3 ± 4.5 c
Pisang rotan	0.13 ± 0.00 a	7.4 ± 0.8 a	0*	131.4 ± 4.9 b
Huwundu vita	0.14 ± 0.00 ab	9.5 ± 0.4 bc	156.8 ± 17.4 a	131.2 ± 3.3 bc
Gabah gabah	0.15 ± 0.00 b	9.7 ± 0.3 bc	169.2 ± 14.2 a	125.7 ± 2.1 bc
Saing hil	0.19 ± 0.00 c	10.1 ± 0.2 c	169.2 ± 6.3 a	110.0 ± 1.8 a
Morong Princessa	0.15 ± 0.00 ab	10.1 ± 0.5 bc	182.7 ± 22.1 ab	124.8 ± 2.9 b
Calcutta 4	0.18 ± 0.01 c	8.4 ± 0.3 ab	151.2 ± 8.3 a	168.5 ± 4.0 d
Kibuzi	0.17 ± 0.00 bc	7.2 ± 0.3 a	264.5 ± 16.8 b	102.8 ± 3.7 a
Kayinja	0.18 ± 0.00 c	13.6 ± 0.4 d	252.2 ± 10.6 b	150.1 ± 3.5 c
Mean	0.16 ± 0.00	9.6 ± 0.1	190.5 ± 4.4	129.8 ± 1.5
F (11,264)	14.589	31.040	8.596	37.103

Data represent means from the assessed genotypes with their standard error (se), Means with different letters within a column are significantly different at (P<0.001), 0* no sucker recorded.

The local EAHB check Kibuzi had the tallest sucker at flowering (264.5 cm), and wild type Calcutta 4 had the shortest (151.2 cm). Diploid Pisang rotan had no follower sucker at flowering, unlike the other diploids. The mean height of the tallest sucker at flowering for Calcutta 4 was significantly different from Kibuzi and Kayinja, but not from the rest.

In general, bunches of triploids weighed more than diploids. Kayinja had the heaviest bunch (19.8 kg), followed by Kibuzi (14.7 kg). They also had the largest finger circumferences (12.7 cm and 13.5 cm, respectively) (**Table 7**). Among the ITC diploids, the lowest mean bunch weight (5.8 kg) was recorded for Pisang rotan, whereas the highest (13.5 kg) was recorded for Pisang gigi buaya. Heavier genotypes generally had longer finger lengths (>10 cm) and circumferences >8.0 cm compared with lighter ones. Diploid Pisang rotan had the second highest number of clusters (9.0) but weighed among the least (5.4 kg), whereas diploid Morong datu had only 4.6 clusters yet weighed 8.0 kg. Diploid Morong datu had the fewest fingers per bunch (80.7), followed by Calcutta 4 (98.8), but weighed eight times more than Calcutta 4. Gabah gabah had the highest number of fingers per bunch (170), followed by Kayinja (155.2), though it weighed less than Kayinja. In terms of bunch weight, triploid clones outperformed all diploids. However, all outsourced diploids from ITC performed better than Calcutta 4 in most agronomic and yield traits.

**Table 7:** Yield performance recorded on the banana bunches of the different genotypes at harvest.

Genotype	Bunch weight (kg) ± se	No. of clusters ± se	Number of fingers ± se	Finger circumference (cm) ± se	Finger length (cm) ± se
Morong datu	8.0 ± 0.8 bc	4.6 ± 0.2 a	80.7 ± 7.4 a	9.6 ± 0.4 c	17.6 ± 0.5 d
Pisang gigi buaya	13.5 ± 1.0 c	8.2 ± 0.2 c	145.2 ± 9.0 bc	9.0 ± 0.2 c	17.3 ± 0.4 d
Pisang tunjuk	11.8 ± 2.7 bcd	7.6 ± 0.2 bc	117.1 ± 6.2 ab	9.3 ± 0.2 c	14.6 ± 0.4 c
SH-3142	7.5 ± 0.5 b	9.4 ± 0.3 cd	167.4 ± 7.3 c	7.6 ± 0.1 b	12.59 ± 0.4 b
Pisang rotan	5.8 ± 0.9 a	9.0 ± 0.3 cd	105.9 ± 3.4 ab	6.8 ± 0.4 ab	15.8 ± 0.9 cd
Huwundu vita	11.9 ± 1.0 c	7.8 ± 0.3 bc	135.8 ± 9.9 bc	8.5 ± 0.1 c	18.2 ± 0.5 d
Gabah gabah	13.0 ± 0.9 c	8.6 ± 0.3 cd	170.3 ± 7.5 c	8.7 ± 0.2 c	16.6 ± 0.6 cd
Saing hil	7.4 ± 0.4 b	6.7 ± 0.2 b	120.7 ± 5.2 b	9.7 ± 0.1 d	11.1 ± 0.2 b
Morong Princessa	7.8 ± 0.6 bc	6.7 ± 0.2 b	99.8 ± 5.5 ab	8.7 ± 0.2 c	16.8 ± 0.5 cd
Calcutta 4	1.0 ± 0.1 a	6.0 ± 0.4 ab	98.8 ± 7.4 ab	5.5 ± 0.3 a	6.5 ± 0.4 a
Kibuzi	14.7 ± 1.1 cd	6.9 ± 0.3 b	100.6 ± 5.8 ab	13.5 ± 0.3 e	15.6 ± 0.4 cd
Kayinja	19.8 ± 1.0 d	10.0 ± 0.4 d	155.2 ± 6.7 c	12.7 ± 0.2 e	16.2 ± 0.3 cd
Mean	10.5 ± 0.5	7.7 ± 0.1	128.9 ± 2.7	9.5 ± 0.1	13.9 ± 0.2
F (11,264)	17.745	21.454	12.693	95.894	70.110

Data represent means ± standard error (se). Means with different letters within a column are significantly different at (P<0.001).

The agronomic performance of the genotypes clearly fit the diploid target profile despite growing in a weevil hot spot. The very low weevil damage in resistant genotypes did not have much effect on agronomic performance.

### 3.5 Performance of the genotypes for pollen quantity

**Figure 2:**
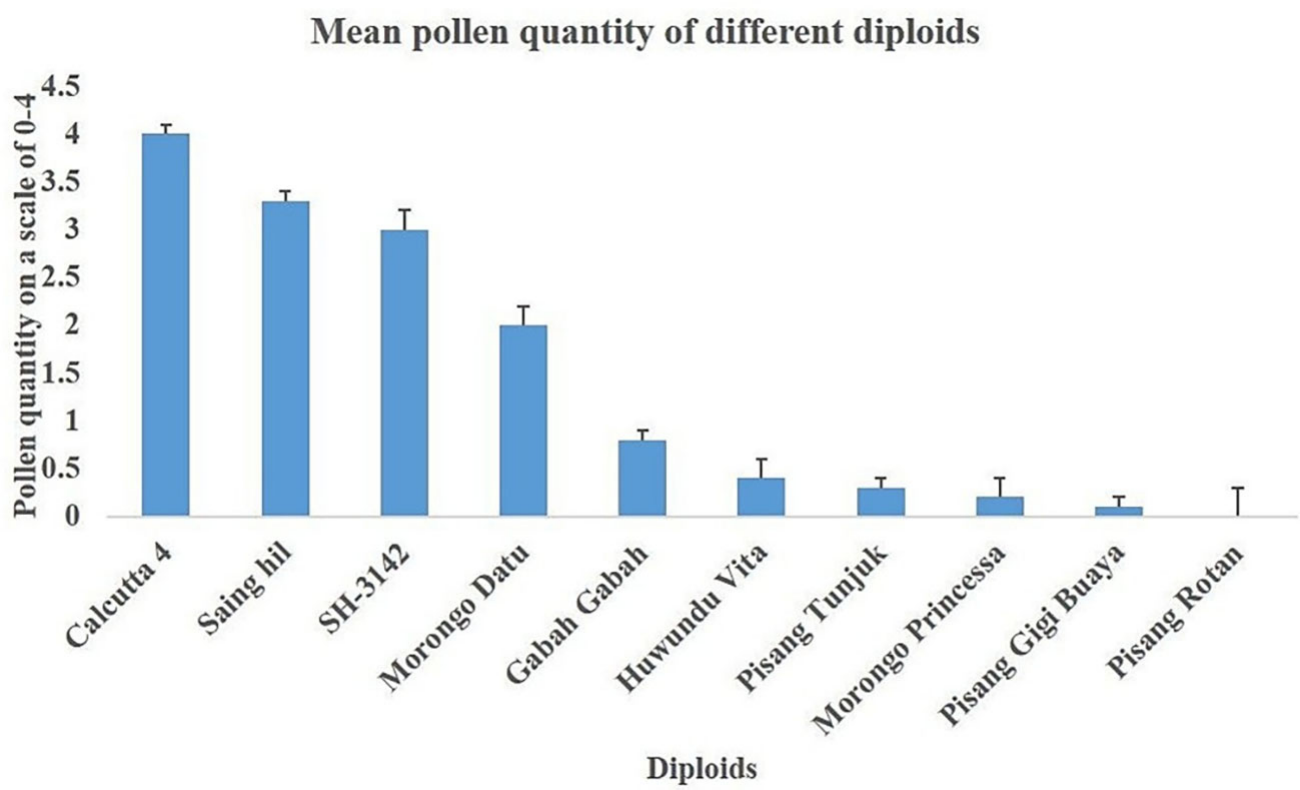
Mean (± se) pollen quantity of the diploids.

The overall mean pollen quantity was 1.2 ± 0.2, with the highest mean recorded in Calcutta 4 (4.0 ± 0.1), whereas the lowest (0.1 ± 0.1) was recorded in Pisang gigi buaya (**Figure 2**). Banana diploids with high pollen quantity were Saing hil, Morong datu, and SH-3142, which were closest to Calcutta 4 in terms of quantity. Other diploids evaluated had very low pollen quantity (<2), while Pisang rotan had no pollen. Genotypes with pollen values below 2 were considered too low for use in crossing.

## 4 Discussion

The significant differences observed among genotypes for the studied traits, such as number of larvae, cross-sectional, peripheral, and overall corm damage, indicate that the genotypes had varying resistance levels to weevil damage. The genotypes that showed high resistance to weevil damage, such as Saing hil, Pisang rotan, Huwundu vita, Morong princessa, Morong datu, Pisang gigi buaya, Pisang tunjuk, and Gabah gabah, had low damage and were comparable to the resistant control and distinguishable from the susceptible. This result agrees with Kiggundu et al. (2003a), who evaluated different banana clones in their study and recommended that diploids were the most important source of resistance to weevils in bananas. The reduced peripheral and cross-sectional corm damage in the resistant genotypes could be due to plant defense mechanisms such as corm hardness, unpalatability, or antibiosis factors (Kiggundu et al., 2006; Arinaitwe et al., 2015). Banana breeding programs integrate resistant genotypes as a source of genes for weevil resistance in susceptible landraces. These should be integrated into the breeding program as a source of genes for weevil resistance in susceptible landraces.

Based on the peripheral and cross-sectional damage, all diploids were resistant to weevils in the field experiment, distinguishable from the susceptible EAHB check. Peripheral and cross-sectional weevil damage are important parameters to evaluate for resistance. Peripheral damage affects root initiation and development, reduces corm diameter, and encourages entry of parasitic microfungi and bacteria, which together affect plantation lifespan. Cross-sectional damage interferes with water, mineral, and food uptake by the plant (Kiggundu et al., 2003; Sadik et al., 2010). The strong correlations between weevil damage on the periphery and cross section in the pot experiment suggest that either peripheral or cross-sectional damage can be used to determine weevil resistance instead of evaluating both. Using a single trait to assess weevil damage saves time and resources during mass screening of genotypes.

The positive and significant correlation between number of larvae and both peripheral and cross-sectional corm damage explains that larval feeding increased damage in susceptible genotypes. The larvae are aggressive feeders, boring through the palatable (susceptible) corms more easily than the resistant genotypes (Night et al., 2011). The highest number of larvae recorded in a susceptible EAHB control indicated that it provided a favorable environment for weevils to breed and multiply. Susceptible banana genotypes show host plant factors that favor weevil survival and fecundity (Night et al., 2010).

This study found no significant difference in the number of weevils recovered among genotypes despite variation in corm damage, as also reported by Sadik et al. (2010) and Twesigye et al. (2018). This is because weevils tend to hide, making their recovery difficult. The number of weevils recovered in pots does not measure resistance or susceptibility accurately in pot experiments. The nylon net creates a microclimate that may affect adult weevils a few days after infestation and hatching. Nevertheless, this does not affect their oviposition and fecundity (Kiggundu et al., 2006). Higher weevil damage for genotype SH-3142 in screen house and field experiments, distinguishable from the susceptible control, categorized it as intermediate. Further evaluation of this genotype under field conditions for at least 5 years will reveal its true response to weevils. According to Gold et al. (2004), weevil damage increases with cycles in susceptible genotypes and is attributed to weevil population build-up over time.

Strong and significant correlations between results from the field and screen house experiments imply that the screen house experiment is reliable in predicting weevil damage in the field. Furthermore, results from the field were in agreement with those from the pot experiment in categorizing genotypes as resistant to weevils, meaning either pot or field experiments can be used to predict resistance. Using pot experiments saves time and resources compared to the long periodic field experiments. They also occupy little space and are cost-effective in mass screening of breeding lines. Natural calamities such as strong winds, hailstorms, and floods are unlikely to affect pot experiments compared with field trials.

In banana breeding, wild and cultivated diploids are used as male parents, and their selection as breeding materials is based on pollen attributes (Ssali et al., 2012). Pollen quantity directly signifies pollen availability for use in genetic improvement of the genetically uniform East African Highland bananas. Thus, diploids with pollen can be incorporated into breeding programs (Ssali et al., 2012; Kitavi et al., 2016). Genotypes Saing hil and SH-3142 recorded high quantities of pollen comparable to Calcutta 4. Further evaluation of pollen viability will reveal their true importance and applicability in banana breeding. Pollen germination studies and staining protocols are valuable in assessing the usefulness of pollen quantity. Genotypes also responded differently in agronomic and yield traits under field conditions. All diploids evaluated showed larger bunch sizes, more clusters, greater plant stature, and earlier maturity, which fall within the acceptable range of the diploid target profile. The good agronomic traits portrayed imply that the adaptability of the imported diploids to Ugandan environmental conditions is possible. Genotype attributes such as plant stature indicate plant stability and resistance to toppling during strong winds. Coupled with resistance to weevils, genotypes Morong princessa, Gabah gabah, and Saing hil had a stature >0.15, recommended for the target diploid profile. Plant height and girth contributed to plant stature; banana genotypes with large girths and short pseudostems are the most preferred by farming communities for easy harvesting. Other attributes such as early maturity are desirable for reducing the long cropping cycle of banana. Genotypes Morong princessa and Morong datu outperformed other diploids in the number of days from flowering to harvest. Furthermore, the number of functional leaves predicts resistance to black Sigatoka and supports photosynthesis, which contributes to yield. All genotypes evaluated performed well compared with the target diploid profile. Banana yield traits such as large fingers, big bunches, and long fingers are preferred for raw banana use, whereas short fingers, small bunches, and small pseudostems are not preferred (Arinaitwe et al., 2014; Akankwasa et al., 2021). All genotypes had bunches >5 kg comparable to Calcutta 4, suggesting a high chance of yield traits being inherited in hybrids. Diploids with multiple desirable traits are preferred, as there are higher chances of heritability of these traits in the final product (Arinaitwe et al., 2015).

We observed that Pisang rotan lacked a follower sucker through the cropping cycle; this is an undesirable trait in banana breeding. To establish a new plantation and ensure ratoon continuity, vegetatively propagated suckers are required. Height of the tallest sucker predicts continuity of the plantation lifecycle. The absence of suckers in Pisang rotan could be a genetically related trait; its multiplication rate in tissue culture under controlled conditions was also much lower compared with the rest of the genotypes.

The number of clusters on the bunch does not necessarily reflect bunch size in diploids. For example, Morong datu and Kibuzi, despite having fewer clusters, weighed 8 and 14 times more, respectively, than Calcutta 4. Thus, number of clusters per bunch is not an important yield trait to consider when selecting diploids.

## 5 Conclusion

Combining large bunch size, large finger size, short maturation period, high pollen quantity, large plant stature, and resistance to weevils, diploid Saing hil should readily be introgressed into the banana breeding program as the male pedigree. Pisang rotan scored a total damage of 0.3, which was statistically different from the resistant check Calcutta 4 (0.2). Genotype SH-3142 had a total damage score of 2.2, which was also statistically different from Calcutta 4. All the other introduced diploid genotypes—Pisang gigi buaya, Pisang tunjuk, Morong princessa, Saing hil, Huwundu vita, Morong datu, and Gabah gabah—had total damage scores between 0.8 and 1.8, which were not statistically different from the resistant check Calcutta 4 and the tolerant diploid SH-3142.

All the screened diploids recorded excellent agronomic traits such as large bunch size, large finger size, short maturity period, high pollen content, and large plant stature. Therefore, the introduced diploids had good weevil resistance traits and can be used as sources of resistance to be introgressed into susceptible EAHBs without greatly affecting agronomic traits.

## 6 Limitation

This study had two major limitations. The first was the subjectivity of pollen quantity data, which could be improved by using pollen-counting methods such as a hemocytometer and other optical density tools to obtain reproducible results. Future pollen studies should also consider pollen viability as a key indicator of fertility.

The second limitation was the absence of non-inoculated controls in the pot and field experiments, which would otherwise have provided a comparable outlook of the genotypes in non-weevil-infested conditions.

## Data Availability

The raw data supporting the conclusions of this article will be made available by the authors, without undue reservation.
